# Recurrent sigmoid volvulus after robotic-assisted laparoscopic prostatectomy

**DOI:** 10.1016/j.eucr.2025.102988

**Published:** 2025-02-21

**Authors:** Kunind Oberoi, Kapil Sethi

**Affiliations:** a Department of Surgery, The University of Melbourne, Parkville, VIC, Australia; b Department of Urology, St Vincent's Hospital Melbourne Pty Ltd, Fitzroy, VIC, Australia

**Keywords:** Sigmoid volvulus, Prostatectomy, Prostate cancer, Laparoscopic, Robotic surgery

## Abstract

A 73-year-old man underwent uncomplicated robotic-assisted laparoscopic prostatectomy (RALP). Nineteen days later, he developed constipation, obstipation, and distension. Imaging confirmed sigmoid volvulus, and he was subsequently managed with endoscopic detorsion. Recurrence occurred two months later, requiring colectomy.

Sigmoid volvulus has not been reported after RALP. RALP may contribute to sigmoid volvulus due to operative patient positioning, pneumoperitoneum, and mesenteric mobilization. Clinicians should be aware of this potential complication, particularly in elderly patients with predisposing anatomical factors. Consideration may be needed to laterally repair any sigmoid released during dissection in RALP.

## 1 Introduction

Sigmoid volvulus is a condition characterized by torsion of the sigmoid mesentery around its pedicle, leading to a closed-loop obstruction. It accounts for 2 %–5 % of all large bowel obstructions in Western countries.^1^ Contributing factors to its development include a ‘longer-than-wide’ mesentery (dolichomesentery), narrowing of the sigmoid mesenteric base, and redundancy of the sigmoid colon—pathological changes commonly associated with aging. Other risk factors include living at high altitudes (>5000 feet), chronic constipation and a high-fiber vegetable diet.^1^^,^^2^

Robot-assisted laparoscopic prostatectomy (RALP) is a widely performed procedure for clinically localized prostate cancer, surpassing open prostatectomy in popularity. In England, RALP accounted for 85.1 % of prostatectomies, and in the United States, 78.1 % between 2005 and 2017.^3^ Compared to open prostatectomy, RALP offers benefits such as reduced blood loss, quicker recovery, shorter hospital stays, less post-operative pain, and lower complication rates. Despite its overall low complication rate, RALP may still lead to minor bleeding, lymphocele, erectile dysfunction, urinary incontinence, postoperative transient ileus, and intestinal injuries such as colorectal perforation.^4^ However, the association between RALP and unusual postoperative complications, such as recurrent sigmoid volvulus remains poorly documented.

Our report aims to present the first case to our knowledge of recurrent sigmoid volvulus following RALP and educate clinicians about the possibility of unusual bowel complications following this urological procedure proposing a pathophysiology on how this complication may have occurred and risk factors that could have facilitated the development of such complication.

## 2 Case presentation

### 2.1 Patient background

Our patient was a 73-year-old male diagnosed with localized prostatic cancer on a background of long-standing metabolic syndrome, osteoarthritis, peptic ulcer disease and two previous cerebrovascular accidents (CVA). His regular medications were aspirin, telmisartan, rosuvastatin, esomeprazole, empagliflozin and metformin. He had no known allergies. He was an ex-smoker and drank one bottle of wine a night.

### 2.2 Prostate cancer diagnosis

The patient was referred to urology with an elevated PSA of 15.4 ng/ml, which was an increase from 8.13 ng/ml performed the year prior. Multiparametric MRI (mpMRI) demonstrated no lesion and a transperineal prostate biopsy (TPB) revealed Gleason 9 (G4+5), grade group 5 prostate cancer of the right posterior lobe. A staging PSMA PET scan confirmed high local prostate avidity and no metastasis. The final histopathology demonstrated a pT3a stage cancer with clear surgical margins.

### 2.3 RALP and discharge

RALP was performed under general anesthetic in the 27-degrees trendelenburg position with a zero-degree telescope. A 24F rectal tube and 18F indwelling catheter (IDC) were inserted during the procedure. Hasson port was passed supraumbilically and insufflated to 10 mmHg with the remaining ports inserted under vision. The sigmoid colon was minimally released laterally to drop the bowel away from the working peritoneal space. There were no adhesions observed and the sigmoid was normal in appearance and attachment. The space of retzius was accessed and a routine prostatectomy was performed. Bladder neck was spared, bilateral nerve sparing was not performed, dorsal venous complex was cut and oversewn. Prior to vesicourethral anastomosis a negative rectal leak test with 60ml air was performed via a syringe, with insufflated air then removed and the rectal tube left open at the end till removal at case completion. The anastomosis was watertight with a negative 180 ml leak test. Total operative time was 160 minutes and estimated blood loss measured at 250 ml. The patient was discharged after two days and postoperative care included analgesia on demand, laxatives for three-days and enoxaparin venous thromboembolism (VTE) prophylaxis.

### 2.4 ED presentation

19-days following RALP the patient presented to the emergency department (ED) with a three-day history of obstipation and reduced oral intake. He also presented with a two-day history of nausea, one episode of vomiting and back pain relieved by lying supine. He denied any abdominal pain, urinary symptoms, fevers or chills. He also denied any recent opioid use and was using paracetamol when required following RALP.

On examination his vitals were normal. His abdomen appeared very distended, however non-tender to light and deep palpation. There was no guarding or peritonism. Bowel sounds were hyperactive on auscultation. Digital rectal examination revealed an empty rectum with no blood or fecal matter.

### 2.5 Sigmoid volvulus diagnosis & management

**Fig. 1:**
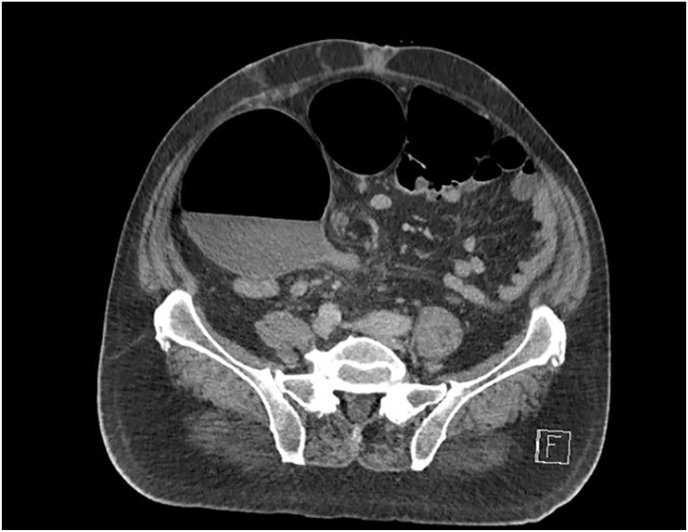
Sigmoid Volvulus Axial CT abdomen/pelvis.

**Fig. 2:**
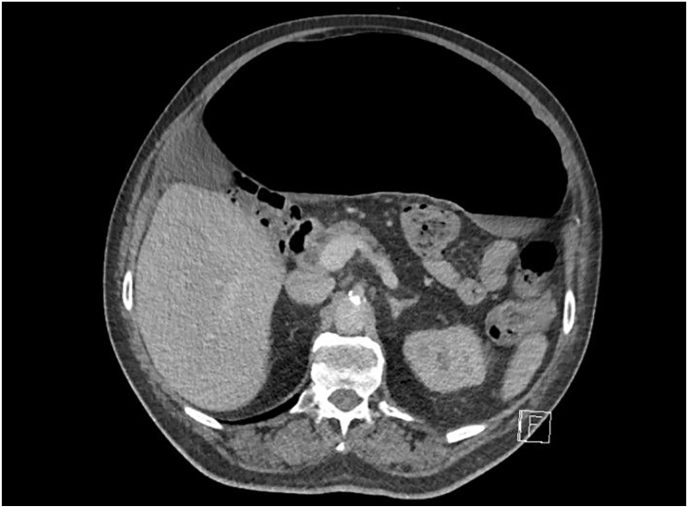
Sigmoid Volvulus Axial CT abdomen/pelvis.

**Fig. 3:**
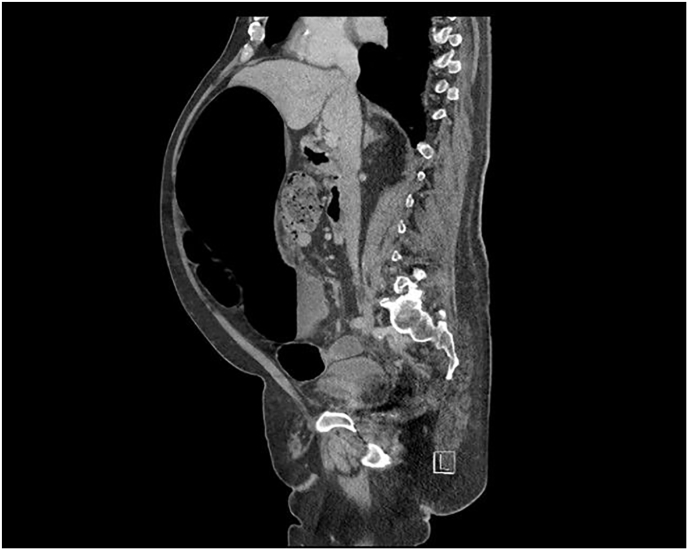
Sigmoid Volvulus Sagittal CT abdomen/pelvis.

**Fig. 4:**
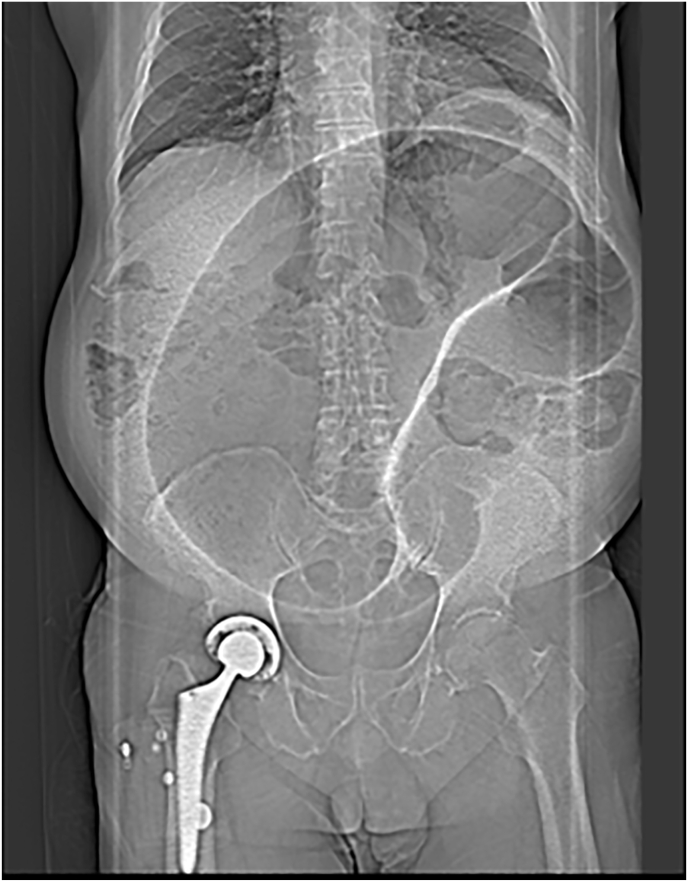
Sigmoid Volvulus anterior/posterior CT abdomen/pelvis.

The patient was admitted, and an abdominal & pelvis contrast-enhanced CT was performed revealing volvulus of the sigmoid colon (Fig. 1, Fig. 2, Fig. 3, Fig. 4). Flexible sigmoidoscopy confirmed a sigmoid volvulus 30 cm of insertion of the scope. There was no masses or blood, and the mucosa appeared normal and healthy. The patient underwent sigmoid detorsion and the colon was decompressed with insertion of a rectal tube. There were no procedural complications during the flexible sigmoidoscope. Postoperatively, he was commenced on regular metoclopramide, cetirizine, analgesia and osmotic laxatives. Postoperative stay was complicated by euglycemic ketoacidosis thus, seven days later the patient was discharged.

Two months after discharge, the patient presented again to ED with a similar three-day history of obstipation, progressive abdominal distension, reduced oral intake for two days, and dull generalized abdominal pain worse centrally. On examination his abdomen was very distended again, bowel loops were palpable and there was mild generalized tenderness with no guarding or rigidity.

Abdominal & pelvis contrast-enhanced CT revealed another sigmoid volvulus, thus the decision was made to perform flexible sigmoidoscopy, detorsion and decompression of the volvulus and insertion of the rectal tube again. One month following his second episode of sigmoid volvulus, an elective anterior resection of the sigmoid colon was performed to prevent recurrence of symptoms following his urological procedure. No operative comment was made about abnormal clinical findings such as a narrow mesentery or large redundant sigmoid colon.

## 3 Discussion

We present a case of a multimorbid obese 73-year old male presenting with recurrent sigmoid volvulus following a recent RALP for localized pT3a, Gleason 9 (G4+5) prostate cancer of the right posterior lobe. His first episode of sigmoid volvulus occurred within a month of his uncomplicated urological procedure with minimal lateral sigmoid mesenteric mobilization to make space during the procedure.

Though bowel complications such as colonic perforation and ileus following RALP are well documented, cases of sigmoid volvulus occurring following RALP have not been reported in the existing literature.

However, the occurrence of cecal volvulus following urological procedures has been discussed sparsely, a review of literature revealed six reported cases of cecal volvulus following radical nephrectomy.^5^, ^6^, ^7^, ^8^, ^9^, ^10^ Only one of these cases was performed laparoscopically and was also performed contralaterally to the cecal volvulus.^5^ Though this data is limited and only makes the association with cecal volvulus, this evidence suggests that there may be a relationship between urological surgery and postoperative sigmoid volvulus. Gonzalez-Urquijo et al. highlighted the association between incising the white line of Toldt to mobilize the colon and the development of cecal volvulus.^10^ The same principle can be applied during lateral sigmoid colon release, where the peritoneal attachment may be lost and the sigmoid becomes more mobile. When surgically detorting the cecal volvulus, they closed the lateral peritoneum which they attribute to no further recurrence of symptoms following retroperitoneal surgery.^10^ Thus, repairing incised mesentery may reduce the likelihood of volvulus occurring following retroperitoneal surgery.

Pneumoperitoneum during laparoscopic surgery combined with risk factors such as an abnormally mobile bowel may move bowel in a position where volvulus may develop.^11^ Thus, it is possible that insufflation of our patient's abdomen during RALP may have allowed excessive movement of bowel intraoperatively leading to the development of volvulus when pneumoperitoneum was withdrawn. Another possible contributing factor for sigmoid volvulus may be the intraoperative positioning of the patient. In literature, lateral tilt during laparoscopic procedures have been discussed as a contributing factor to postoperative volvulus.^11^ Our patient who underwent RALP was placed supine in the 27-degrees trendelenburg position. We suggest that placing our patient in this position may have led to his sigmoid colon, especially if redundant, becoming more mobile predisposing him to developing volvulus postoperatively. Placing our patient in this position, combined with dissection and lateral mobilization during his RALP may have narrowed and lengthened the sigmoid mesentery resulting in the recurrent postoperative sigmoid volvulus.

Previous abdominal surgery can contribute to postoperative adhesions, creating a rotation axis that increases the risk of volvulus.^12^ A review of laparoscopic cholecystectomy cases found that 50 % of postoperative volvulus patients had prior abdominal surgeries such as hysterectomy, appendectomy, or caesarean section.^13^ Though our patient had no history of abdominal surgery, existing literature suggests these factors can increase the likelihood of postoperative volvulus.

Anatomical predisposition may also play a role. In cecal volvulus, 50 % of patients report 'mobile cecal syndrome' characterized by intermittent right lower quadrant pain and abdominal distension relieved by passing flatus prior to developing volvulus.^14^ A redundant sigmoid colon with an elongated mesentery and a narrow base can be congenital but is more commonly an acquired condition associated with factors such as high altitude, aging, chronic constipation, and high-fiber diets.^1^

Sigmoid volvulus typically presents around age 55, with individuals over 60 experiencing increased sigmoid redundancy and higher recurrence rates.^1^^,^^2^^,^^15^^,^^16^ Elderly patients are more prone to volvulus recurrence than younger individuals due to anatomical changes associated with aging.^1^^,^^2^^,^^15^^,^^16^

Dietary factors also contribute. High-fiber diets increase the risk of sigmoid volvulus more than chronic constipation, as the increased bulkiness of stool from undigested fiber leads to fecal loading, distension, and elongation of the sigmoid colon.^17^ However, habitual constipation can also contribute by gradually elongating the sigmoid, which may explain its association with volvulus recurrence, particularly in elderly patients or those with neurologic, psychiatric, or metabolic conditions.^1^^,^^2^

Nevertheless, patients with congenital or acquired sigmoid redundancy may have an increased risk of volvulus following laparoscopic surgery. Unlike 'mobile cecal syndrome,' sigmoid redundancy is usually asymptomatic until volvulus occurs, making it difficult to identify at-risk individuals preoperatively.^1^ However, screening for potential risk factors may help assess the likelihood of developing sigmoid volvulus after RALP.

## 4 Conclusion

In this case we reported the presentation of recurrent sigmoid volvulus following RALP. This case is a new addition to the wide range of reported bowel complications following RALP. After exclusion of more benign causes of intestinal obstruction, sigmoid volvulus may occur in patients who undergo lateral peritoneal release to mobilize the sigmoid colon. Consideration may be needed to laterally repair any sigmoid released during dissection in RALP.

## CRediT authorship contribution statement

**Kunind Oberoi:** Conceptualization, Investigation, Methodology, Writing – original draft, Writing – review & editing. **Kapil Sethi:** Conceptualization, Investigation, Methodology, Supervision, Writing – review & editing.

## Consent

Informed consent was duly obtained for the publication of this case series report and any supplementary images.

## Funding sources

This research did not receive any specific grant from funding agencies in the public, commercial, or not-for-profit sectors.

## Conflicts of interest & financial disclosures

None.

## Glossary:

RALP: Robotic assisted laparoscopic prostatectomy
PSA: prostate specific antigen
PSMA-PET: prostate-specific membrane antigen positron emission tomography
MRI: Magnetic resonance imaging
